# AUTS2 Syndrome: Molecular Mechanisms and Model Systems

**DOI:** 10.3389/fnmol.2022.858582

**Published:** 2022-03-31

**Authors:** Alecia Biel, Anthony S. Castanza, Ryan Rutherford, Summer R. Fair, Lincoln Chifamba, Jason C. Wester, Mark E. Hester, Robert F. Hevner

**Affiliations:** ^1^The Steve and Cindy Rasmussen Institute for Genomic Medicine, Abigail Wexner Research Institute at Nationwide Children’s Hospital, Columbus, OH, United States; ^2^Department of Pathology, University of California, San Diego, San Diego, CA, United States; ^3^Department of Neuroscience, The Ohio State University College of Medicine, Columbus, OH, United States; ^4^Department of Pediatrics, The Ohio State University College of Medicine, Columbus, OH, United States

**Keywords:** intellectual disability, microcephaly, RNA-binding protein, AUTS2 syndrome, FBRSL1, dentate gyrus hypoplasia, cerebellar hypoplasia, cerebral organoids

## Abstract

AUTS2 syndrome is a genetic disorder that causes intellectual disability, microcephaly, and other phenotypes. Syndrome severity is worse when mutations involve 3’ regions (exons 9-19) of the *AUTS2* gene. Human AUTS2 protein has two major isoforms, full-length (1259 aa) and C-terminal (711 aa), the latter produced from an alternative transcription start site in exon 9. Structurally, AUTS2 contains the putative “AUTS2 domain” (∼200 aa) conserved among AUTS2 and its ohnologs, fibrosin, and fibrosin-like-1. Also, AUTS2 contains extensive low-complexity sequences and intrinsically disordered regions, features typical of RNA-binding proteins. During development, *AUTS2* is expressed by specific progenitor cell and neuron types, including pyramidal neurons and Purkinje cells. AUTS2 localizes mainly in cell nuclei, where it regulates transcription and RNA metabolism. Some studies have detected AUTS2 in neurites, where it may regulate cytoskeletal dynamics. Neurodevelopmental functions of AUTS2 have been studied in diverse model systems. In zebrafish, *auts2a* morphants displayed microcephaly. In mice, excision of different *Auts2* exons (7, 8, or 15) caused distinct phenotypes, variously including neonatal breathing abnormalities, cerebellar hypoplasia, dentate gyrus hypoplasia, EEG abnormalities, and behavioral changes. In mouse embryonic stem cells, AUTS2 could promote or delay neuronal differentiation. Cerebral organoids, derived from an AUTS2 syndrome patient containing a pathogenic missense variant in exon 9, exhibited neocortical growth defects. Emerging technologies for analysis of human cerebral organoids will be increasingly useful for understanding mechanisms underlying AUTS2 syndrome. Questions for future research include whether AUTS2 binds RNA directly, how AUTS2 regulates neurogenesis, and how AUTS2 modulates neural circuit formation.

## Introduction to AUTS2 Syndrome

The *AUTS2* gene (autism-susceptibility-gene-2) was first identified in humans by genetic analysis of monozygotic twins with autism and chromosomal translocation *t*(7;20) (Sultana et al., 2002). Human *AUTS2* was further revealed as a 1.2-Mb gene on chromosome 7q11.22, which encodes a 1259-aa full-length protein, and a 711-aa C-terminal isoform (Beunders et al., 2013). Sequence analysis of AUTS2 detected motifs such as proline-rich regions and histidine repeats, but no recognizable structural domains. As of 2021, more than 60 patients with pathogenic *AUTS2* variants have been reported, and the AUTS2 syndrome has been well characterized as a neurodevelopmental and somatic malformation disorder with diverse phenotypes. The most common phenotypes are intellectual disability (ID) and microcephaly (Beunders et al., 2016).

Although the *AUTS2* gene was named for autism susceptibility, many AUTS2 syndrome patients have an outgoing personality in childhood (Beunders et al., 2016). Rather, the most frequent trait is ID (or developmental delay), mild to severe, in virtually all patients. The major traits and their frequency are ID (98%), microcephaly (65%), feeding difficulties (62%), attention deficit hyperactivity disorder (ADHD) (54%), and autistic traits (52%) (Beunders et al., 2013, 2016; Sanchez-Jimeno et al., 2021). Hypotonia (38%) and spasticity (37%), inverse disorders of neuromuscular reflexes, are also relatively frequent. A small minority have epilepsy (7%). As observed by neuroimaging, structural brain anomalies occur in 27% of *AUTS2* patients (Sanchez-Jimeno et al., 2021). The reported brain malformations include corpus callosum hypoplasia, cerebellar hypoplasia, small posterior fossa, and Chiari malformation type 1 (Liu et al., 2021; Fair et al., 2022). Somatic developmental problems are also numerous in AUTS2 syndrome. These include growth defects, such as low birth weight (20%) and short stature (43%); musculoskeletal anomalies, such as kyphosis/scoliosis (24%) and tight heel cords (37%); and facial dysmorphisms, such as hypertelorism (44%) and micrognathia/retrognathia (36%). Thus, in the most severe cases, AUTS2 syndrome can affect many organs.

As a gauge of overall severity, an “AUTS2 syndrome severity score” representing the sum of 32 traits was formulated (Beunders et al., 2013). Interestingly, the severity scores were observed more severe in patients with whole gene deletions or C-terminal mutations (exons 9–19), and less severe in patients with N-terminal mutations (exons 1–8) (Beunders et al., 2013; Sanchez-Jimeno et al., 2021). The hypothesis that the C-terminal part of the protein mediates major AUTS2 functions was further supported by *Auts2a* knockdown experiments in zebrafish, in which expression of the C-terminal isoform rescued the microcephaly phenotype (Beunders et al., 2013). The C-terminal isoform is comprised of a proline-rich region (exons 9–13), the putative “AUTS2 domain” of ∼200 aa (exons 14–19), and disordered regions (exon 19), but does not include the HX repeat encoded by the first part of exon 9 (described in more detail below). Indeed, the alternative tss, corresponding to position 1,597 of full-length *AUTS2* cDNA (Beunders et al., 2013), lies within the HX repeat encoded by cDNA positions 1,575–1,626. The translation start site for AUTS2-C corresponds to position 1,666 of full-length cDNA, and thus does not include the HX repeat.

AUTS2 syndrome exhibits phenotypic overlap with several other genetic causes of ID, including Rubinstein-Taybi syndrome (*CREBBP* or *EP300*) (Fergelot et al., 2016), NONO syndrome (Sewani et al., 2020), TBR1 syndrome (Nambot et al., 2020), and FBRSL1 syndrome (Ufartes et al., 2020). The similarities among these disorders reflect their related roles in genetic pathways and binding interactions. Specifically, expression of *Auts2* is regulated by transcription factor TBR1 (Bedogni et al., 2010); AUTS2 protein interacts with NONO protein (Castanza et al., 2021); AUTS2 binds and regulates *EP300* mRNA (Castanza et al., 2021); and FBRSL1, an RNA-binding protein (Baltz et al., 2012), is the closest homolog of AUTS2 in the genome (Sellers et al., 2020).

## The *AUTS2* Gene Family and Functions

When *AUTS2* was first described, its functions could not be predicted on the basis of homology to known proteins. Indeed, only partial sequence homology was found to two genes with unknown functions, designated CG9056 and FLJ11618 (Sultana et al., 2002). Subsequent research identified CG9056 as Drosophila tay bridge (*tay*), and FLJ11618 as human fibrosin (*FBRS*). Both genes, it is now known, indeed belong to the same diversified superfamily of genes as *AUTS2* (Sellers et al., 2020).

More narrowly, the “*AUTS2* gene family” consists of the three most closely related genes, *AUTS2, FBRSL1*, and *FBRS*. These genes are ohnologs—defined as paralogs produced by two-rounds of gene duplication from a single ancestral gene during the evolution of jawed vertebrates ∼450 million years ago (Sacerdot et al., 2018; Sellers et al., 2020). Accordingly, *AUTS2, FBRSL1*, and *FBRS* genes are found in all vertebrates. By sequence analysis, *AUTS2* appears most closely related to the ancestral *AUTS2* precursor (*aAUTS2p*) gene; also, *AUTS2* and *FBRSL1* are closer to each other than to FBRS (Sellers et al., 2020).

The ancestral *aAUTS2p* gene arose in early bilaterian animals ∼650 million years ago, and its derivatives are found in the *Nephrozoa* clade (Sellers et al., 2020). Thus, non-Chordate bilaterians, such as flies and amphioxus, contain only one *AUTS2*-related gene. In Drosophila, that gene is *tay* (tay bridge), which regulates neuronal development in the protocerebral bridge and motoneurons (Poeck et al., 2008; Molnar and de Celis, 2013; Molnar et al., 2018). However, *tay* is relatively divergent from *aAUTS2p*, and shows only patchy homology to mammalian *AUTS2* (Sellers et al., 2020). The most highly conserved sequence in AUTS2-related proteins from diverse vertebrate and invertebrate species aligns with exon 14 of human *AUTS2*, which encodes part of the predicted “AUTS2 domain.” Also highly conserved is the “HX repeat” or “HQHT repeat” region encoded in the first half of *AUTS2* exon 9.

While much progress has been made comprehending evolution of the *AUTS2* gene family, no consensus conserved functions of the proteins have been determined. Several AUTS2 superfamily members localize in cell nuclei, including AUTS2 (Bedogni et al., 2010), FBRSL1 (Ufartes et al., 2020), and tay bridge (Molnar and de Celis, 2013). Among these, FBRSL1 was previously identified as an mRNA binding protein (Baltz et al., 2012). Further studies of the *AUTS2* gene family and superfamily will be necessary to shed light on conserved and divergent functions of these proteins.

## Structure of the Human *AUTS2* Gene, Transcripts, and Protein Isoforms

**FIGURE 1:**
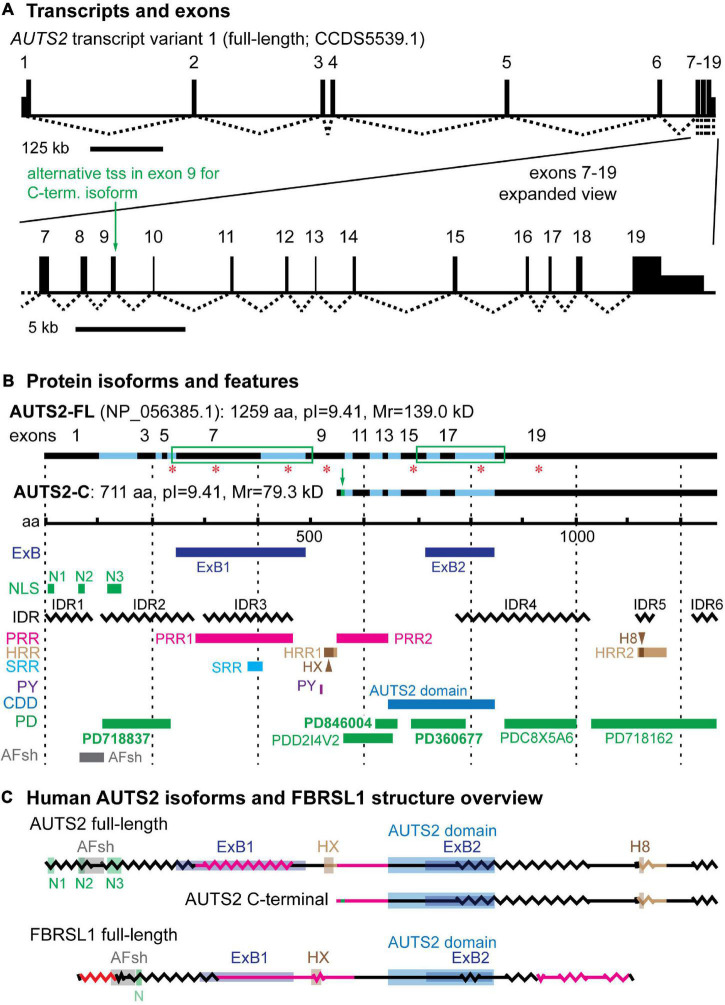
*AUTS2* transcripts and protein isoforms. **(A)** The full-length *AUTS2* transcript has 19 exons. An alternative transcription start site (tss) in exon 9 produces mRNA for the C-terminal protein isoform. **(B)** The full-length and C-terminal isoforms are indicated with source exons in black (odd) and blue (even). Green boxes enclose splice junctions in which amino acids were encoded across the junctions, comprising exon blocs. Red asterisks indicate source exons that encoded a non-integer number of amino acids. Features mapped from protein sequence: ExB, exon bloc; NLS, nuclear localization sequence; IDR, intrinsically disordered region; PRR, Pro-rich repeat; HRR, His-rich repeat; HX, HX repeat; H8, polyhistidine (8×) repeat; SRR, Ser-rich repeat; PY, PY protein binding motif; CDD, conserved domain database; PD, ProDom predicted domains (domain names in bold also identified in FBRSL1); AFsh, AUTS2-FBRSL1 short homology region. **(C)** Both major AUTS2 isoforms have a high content of IDRs (zigzag lines) and regions enriched in amino acids Pro, His, or Ser (red lines). The C-terminal isoform includes the AUTS2 domain, but lacks N-terminal features such as the HX repeat.

Human *AUTS2* spans 1,195,032 base pairs on chromosome 7q11.22 (UCSD Genome Browser, assembly hg38^1^). Exons 1–7 are separated by long introns (16–301 kb), while exons 7–19 have shorter introns (∼1–3 kb) (Figure 1A and Supplementary Table 1). Full-length AUTS2 cDNA (NCBI CCDS5539.1) encodes a protein of 1259 amino acids (NP_056385.1), here designated isoform AUTS2-FL (Figure 1B). Many variant transcripts have been annotated from sequencing projects, but the best documented variant is generated from an in-frame alternative transcription start site (tss) in exon 9, to produce the C-terminal isoform of AUTS2 (Beunders et al., 2013), here designated AUTS2-C (Figures 1A,B). The AUTS2-C transcript also utilizes an alternative splice junction between exons 9–10 to incorporate an additional 7 amino acids, totaling 711 aa. Both AUTS2-FL and AUTS2-C are highly basic (pI = 9.41).

Sequence analysis has revealed several features of AUTS2 protein that suggest possible functions (Figure 1B). Among the most salient features of AUTS2 are its high contents of predicted intrinsically disordered regions (IDRs) and low complexity sequences (LCSs). One recent analysis found that predicted IDRs comprise 64.4% of AUTS2-FL (Sellers et al., 2020). Among LCSs, AUTS2 contains two proline-rich regions (PRRs), two histidine-rich regions (HRRs), and one serine-rich region (SRR) (Sultana et al., 2002; and Expasy ScanProsite) (Figure 1B). HRR1 contains an HX (HQ and HT) repeat (aa 525–542), while HRR2 contains an 8-aa stretch of only histidines. Together, putative IDRs and LCSs cover ∼74.2% of AUTS2-FL. This is significant because IDRs and short repetitive amino acid motifs are typical features of mRNA-binding proteins (Castello et al., 2012; Hentze et al., 2018). Consistent with this analysis, AUTS2 was recently found to bind RNA-protein complexes, and possibly RNA directly (Castanza et al., 2021).

Sequence analysis also indicates that AUTS2 is likely to be localized in cell nuclei. AUTS2-FL has multiple nuclear localization sequences (NLSs) in the 5′ region, and NucPred indicates 100% likelihood of intranuclear localization. AUTS2-C does not have discrete NLSs, but its overall sequence predicts 70% likelihood of intranuclear localization (NucPred). In addition, AUTS2-FL, but not AUTS2-C, contains a PY motif (PPPY), which may bind WW-domains to interact with other proteins. PY motifs interact with Nedd4 family E3 ubiquitin ligases for proteolysis (Hatstat et al., 2021), and with other proteins for signal transduction or transcription (Lin et al., 2019).

The domain structure of AUTS2 is unknown. The protein contains no recognized structural domain sequences, and its structure has not been studied experimentally. Previous studies have used bioinformatics to propose AUTS2-FL structures with three domains (Sellers et al., 2020) or five domains (Castanza et al., 2021), but these remain speculative. Sequence analysis of AUTS2 using NCBI Conserved Domains Database identifies an ∼200-aa “AUTS2 domain” (pfam15336), predicted on the basis of sequence conservation across the *AUTS2* gene family (*AUTS2, FBRSL1*, and *FBRS*) (Castanza et al., 2021). The AUTS2 domain is present in both AUTS2-FL and AUTS2-C isoforms (Figure 1B). Sequence analysis of AUTS2-FL with ProDom identified six possible domains, three of which are also found in FBRSL1 (Supplementary Figure 1 and Supplementary Table 2). All of the putative domains identified by ProDom are annotated as likely poly(A)-RNA binding proteins. Finally, comparison of AUTS2 and FBRSL1 identifies a highly conserved 46 aa sequence (73.9% identity, no gaps) in the N-terminal part of AUTS2-FL, here designated the AUTS2/FBRSL1 short homology (AFsh) region (Figure 1B, Supplementary Figure 1B, and Supplementary Table 3).

Other interesting features of *AUTS2* include two blocs of exons, ExB1 and ExB2 (comprised of exons 6–9 and 15–19, respectively), each connected by splice junctions that encode amino acids across the junctions; as well as several exons that encode non-integer numbers of amino acids (Figure 1B). These features imply reduced likelihood of alternative splicing involving those exons and may indicate important functions for the encoded protein sequences. Indeed, ExB1 encodes PRR1, SRR, and IDR regions, potentially for RNA binding; ExB2 encodes part of the AUTS2 domain. Interestingly, ExB1 and ExB2 are conserved in FBRSL1 as exons 6–9 and 13–17 of that gene (Supplementary Figure 1 and Supplementary Table 3). For ExB1, the encoded protein sequences are relatively divergent between AUTS2 and FBRSL1, mostly due to insertions and deletions involving PRR1. In contrast, ExB2 is better conserved at the protein level, and encodes most of the putative AUTS2 domain in both FBRSL1 and AUTS2.

To further explore potential AUTS2 protein structures, we used RoseTTAfold^2^ (Baek et al., 2021) to computationally predict structures of AUTS2, FBRSL1, and the AUTS2 domains of each protein (Supplementary Figures 2–5). None of the RoseTTAfold models yielded high-confidence results, most likely because no known structures are available for proteins of similar sequence. However, all of the models showed extensive stretches of disordered, open structure. Indeed, one possibility is that outside the putative AUTS2 domain, AUTS2 and FBRSL1 lack classic well-structured domains, and instead utilize disordered sequences for RNA binding. Putting all features together, both AUTS2 and FBRSL1 are seen as proteins with extensive IDRs and LCSs, punctuated by conserved motifs and the putative AUTS2 domain (Figure 1C and Supplementary Figure 1C).

## AUTS2 Expression Patterns and Intracellular Localization

**FIGURE 2:**
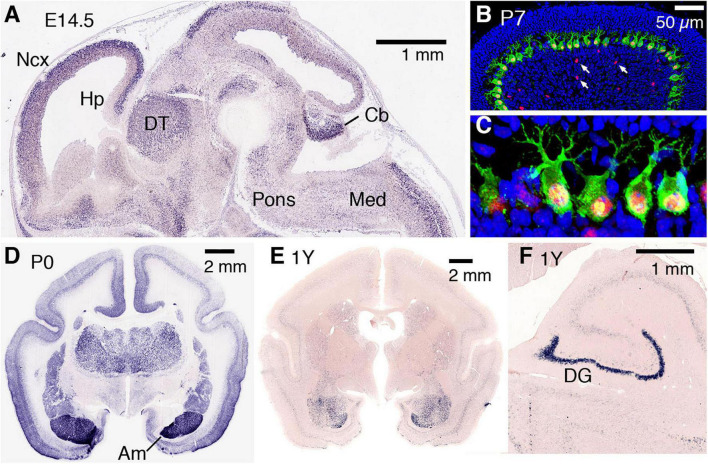
AUTS2 expression in developing mouse and marmoset brain. **(A)** By *in situ* hybridization, Auts2 expression is observed in multiple areas of E14.5 mouse brain, including cerebral neocortex (Ncx), hippocampus (Hp), dorsal thalamus (DT), pons, cerebellum (Cb), and medulla (Med). Sagittal section from Genepaint (https://gp3.mpg.de/). **(B,C)** In P7 mouse cerebellum, AUTS2 protein (red) localizes in the nuclei of calbindin + (green) Purkinje cells, and in scattered Golgi neurons (arrows). Panel **(C)** magnified 5× from **(B)**. **(D)** In neonatal marmosets, AUTS2 is expressed in many brain regions, with highest levels in amygdala (Am). **(E,F)** In 1-year-old (young adult) marmosets, AUTS2 levels remain high in amygdala and dentate gyrus (DG). Data for panels **(D–F)** from the Marmoset Gene Atlas (https://gene-atlas.brainminds.riken.jp/).

The expression of *AUTS2* mRNA and protein isoforms has been studied in multiple tissues and cell types (Table 1). In humans, *AUTS2* mRNA is expressed at relatively high levels in fetal and adult brain, skeletal muscle, and kidney; and at lower levels in several other tissues (Sultana et al., 2002). In mice, *Auts2* mRNA is expressed in many areas of the developing brain and spinal cord (Figure 2A) (Bedogni et al., 2010). In developing cerebral cortex, *Auts2* is expressed mainly in the cortical plate, where postmitotic neurons are located, although lower levels of mRNA are also detected in progenitor compartments (ventricular zone and subventricular zone) (Bedogni et al., 2010). Moreover, AUTS2 protein has been detected in neurogenic cortical progenitor cells (Castanza et al., 2021). From E16.5 to the first postnatal week in mice, *Auts2* mRNA is expressed in an intracortical gradient from high rostral to low caudal, suggesting a possible role in cortical patterning (Bedogni et al., 2010). AUTS2 protein is localized mainly in the nuclei of neurons, but not glial cells; in cerebral cortex, AUTS2 is expressed by pyramidal neurons, but not GABAergic interneurons (Bedogni et al., 2010; Gao et al., 2014; Castanza et al., 2021). AUTS2 protein may additionally be present in neuronal cytoplasm, including neurites and growth cones (Hori et al., 2014). In postnatal mouse cerebellum, AUTS2 protein is detected at high levels in Purkinje cells and Golgi neurons (Figures 2B,C) (Yamashiro et al., 2020).

**TABLE 1:** AUTS2 expression in developing brain and cultured cells.

Tissue/cell type	Auts2 gene expression	AUTS2 isoform expression	References
Embryonic stem cells	Before differentiation (D0)	Long only	Monderer-Rothkoff et al., 2021
	Differentiation day 6 (D6)	Short only	
	D12 (corresponding to ∼E12)	Long and short	
Whole brain	mRNA Peaks at E16, decreases until reaching low levels at P21	Short > long; Both decrease throughout early development	Bedogni et al., 2010; Hori et al., 2014; Hori et al., 2020; Liu et al., 2021
	Early embryonic stages, mRNA strongest expression in neocortex, hippocampus, and cerebellum		
Cerebral cortex	Rostral (high expression)-caudal (low expression) gradient	Short predominate; Low levels of Long	Bedogni et al., 2010; Hori et al., 2014; Castanza et al., 2021
Hippocampus	From E14 onward: dentate gyrus (DG), CA1, and CA3	Predominantly short	Bedogni et al., 2010; Castanza et al., 2021
	P21: granule cell layer and subgranular zone		Bedogni et al., 2010
Cerebellum	Early stages: granule neurons, precursor of Purkinje cells, and some deep nuclei	Long > short	Bedogni et al., 2010; Castanza et al., 2021
	P21: Purkinje cells and Golgi neurons		Bedogni et al., 2010; Yamashiro et al., 2020; Castanza et al., 2021
Thalamus	E14: dorsal thalamus		Bedogni et al., 2010
	P21: anterior thalamic nuclei and ventrolateral/ventromedial nuclei only	Unknown	Bedogni et al., 2010
Fetal brain	8 weeks: frontal, parietal, and temporal lobes of the neocortex, telencephalon, ganglionic eminence, caudate nucleus, putamen nuclei, and cerebellum	Short predominate in early stages (8–24 weeks), both transcripts are expressed in similar low levels in adult brain	Oksenberg et al., 2013; Pinto et al., 2020; Monderer-Rothkoff et al., 2021
	23 weeks: dentate gyrus, CA1 and CA3 pyramidal cell subregions, the ganglionic eminence, caudate nucleus, and putamen nuclei; neocortex and prefrontal cortex		

Recently, the open Marmoset Gene Atlas^3^ has published *AUTS2* expression results in the developing and adult non-human primate brain. As shown by *in situ* hybridization, *AUTS2* mRNA is expressed in many regions of the developing marmoset brain, with particularly high levels in the amygdala (Figure 2D). In adult marmosets, *AUTS2* mRNA levels remain high in the amygdala, and in granule neurons of the hippocampal dentate gyrus (Figures 2E,F).

## Molecular Functions and Interactions of AUTS2 Protein

### Transcriptional Activation

**FIGURE 3:**
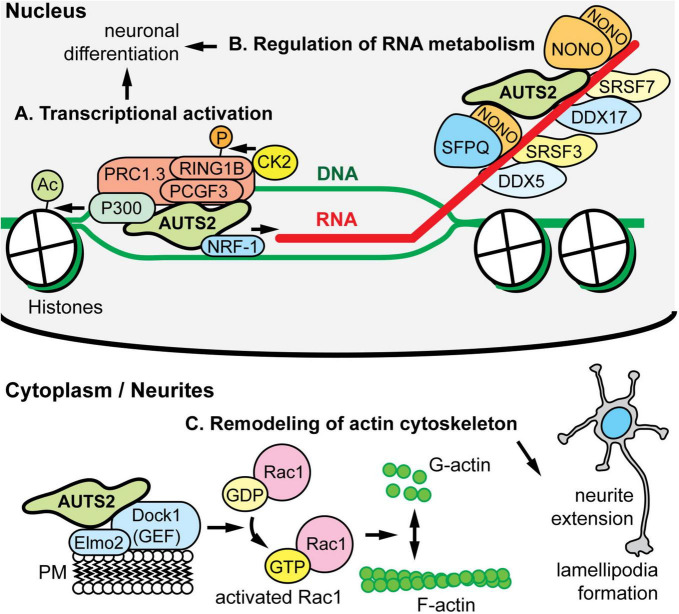
AUTS2 molecular functions and interactions. **(A)** In HEK293 cells, AUTS2 associates with variant forms of polycomb repressive complex 1 (vPRC1), which also contains PCGF3/5, RING1B, and other PRC1 subunits (PMID: 25519132). In turn, AUTS2 recruits P300, a histone acetyltransferase that opens chromatin, and binds NRF1, a transcriptional activator (PMID: 34637754). Also, vPRC1 recruits CK2, a protein kinase that phosphorylates and inactivates RING1B, a ubiquitin ligase and core subunit of PRC1 for chromatin inactivation. The net effect of AUTS2 is to convert PRC1 from a repressor to an activator of transcription. **(B)** In developing cerebral cortex, AUTS2 associates with multiple RNA-binding proteins (RBPs), including the scaffolds NONO and SFPQ; splicing factors SRSF3 and SRSF7; and RNA helicases DDX5 and DDX7 (PMID: 34013328). Also, AUTS2 co-immunoprecipitated multiple RNA species, suggesting that AUTS2 binds RNA directly or indirectly. **(C)** In HEK293 cells, AUTS2 associates with a guanine nucleotide exchange factor (GEF) complex containing DOCK1 (also known as DOCK180) and ELMO2 (PMID: 25533347). Through this interaction, AUTS2 is thought to cause activation of Rac1 (a small G protein), remodeling of the cytoskeleton, neurite elongation, and lamellipodia formation. AUTS2 was also found to interact with another GEF, P-REX1; and may regulate multiple small G proteins.

Molecular functions of AUTS2 have been elucidated in the context of AUTS2 interacting molecules, complexes, and chromatin (Figure 3). The first proposed function of AUTS2, as a transcriptional activator, was determined on the basis of its interactions with other regulators of transcription, and its distribution in active open chromatin. In human embryonic kidney (HEK) cells induced to express AUTS2, AUTS2 associated with non-canonical forms of polycomb repressive complex 1 (PRC1), which is a complex that contains PCGF3/5, RING1A/B, and RYBP/YAF2, but no CBX proteins (Gao et al., 2014). Canonical PRC1 is an epigenetic repressor that ubiquitinates histones. In contrast, non-canonical PRC1 (ncPRC1) was found to activate gene expression via AUTS2-mediated recruitment of P300, a histone lysine acetyltransferase (Figure 3A) (Gao et al., 2014). Consistent with this function, ChIP-seq indicated that in developing brain, AUTS2 localizes to actively transcribed chromatin, usually within ±5 kb of transcriptional start sites (Gao et al., 2014; Oksenberg et al., 2014; Liu et al., 2021). Subsequently, WDR68 was identified as an additional component of the AUTS2-containing ncPRC1 complex (Wang et al., 2018). On the other hand, two groups have reported that AUTS2-FL interacts with ncPRC1, but AUTS2-C does not (Geng et al., 2021; Monderer-Rothkoff et al., 2021). In a study of mutations in AUTS2 syndrome, the interaction of AUTS2 with P300 in HEK 293 cells was found to be disrupted by mutations involving the HX repeat (Liu et al., 2021). Importantly, the HX repeat encompasses the alternative tss for AUTS2-C, and indeed a small deletion in the HX repeat was found to eliminate expression of AUTS2-C (Martinez-Delgado et al., 2021). Significantly, most of the above-mentioned AUTS2-protein interactions and disruptions were studied in HEK cells after AUTS2 overexpression (Gao et al., 2014; Wang et al., 2018; Geng et al., 2021), or in yeast two-hybrid assays (Monderer-Rothkoff et al., 2021). Further studies are needed to confirm that AUTS2 interacts with proteins such as PCGF3/5, RING1B, and P300 at physiological levels in cortical neurons *in vivo*.

### RNA Metabolism

Another proposed intranuclear function of AUTS2 is to regulate RNA metabolism by associating with RNA-binding protein (RBP) complexes, and possibly with RNA directly. Immunoprecipitation (IP) of AUTS2 from developing mouse cortex followed by mass spectrometry (IP-MS) revealed that AUTS2 interacts with multiple RBPs *in vivo*, including scaffolds NONO and SFPQ, splicing factors such as SRSF3/7, and RNA helicases DDX5/17 (Figure 3A) (Castanza et al., 2021). Also, in yeast two-hybrid assays, AUTS2 was reported to interact with splicing factor SF3B1 (Monderer-Rothkoff et al., 2021). In neonatal mouse neocortex, AUTS2 IP followed by RNA sequencing (RIP-seq) detected abundant transcripts, including many, such as *Ep300*, that are dysregulated in *Auts2* conditional mutant mice (described below) (Castanza et al., 2021). The hypothesis that AUTS2 may bind RNA directly is further supported by its high content of IDRs and LCSs, characteristic of RBPs (Castello et al., 2012; Hentze et al., 2018) (Figure 1). Furthermore, FBRSL1—the closest homolog (ohnolog) of AUTS2—has been identified as a poly(A)-RBP (Baltz et al., 2012). More studies of RNA regulation by AUTS2 are needed to determine if AUTS2 interacts directly with RNA, or only indirectly through protein complexes.

### Cytoskeletal Dynamics

Outside the nucleus, AUTS2-FL was found to interact with guanine nucleotide exchange factors (GEFs) such as P-Rex1 and the Dock1/Elmo2 complex (Figure 3B) (Hori et al., 2014). These GEFs activate Rac1, a small G protein “molecular switch” that controls cytoskeletal organization. By interacting with GEFs, AUTS2 was proposed to enhance Rac1 activation and thus control neurite outgrowth, cell migration, and the formation of lamellipodia and filopodia (Hori et al., 2014). For future studies, it would be useful to see if these AUTS2-GEF interactions, found in HEK cells after overexpression, also occur under physiologic conditions in neurons *in vivo*.

### Inhibition of BMP Signaling to Promote Neuronal Differentiation

In HEK cells and in mESCs differentiated to radial glia-like neuronal progenitor cells, both AUTS2-FL and AUTS2-C interacted with WDR68 and SKI to form a novel AUTS2-WDR68-SKI (AWS) complex (Geng et al., 2021). The AWS complex recruited CUL4, a ubiquitin E3 ligase, to mediate proteolytic degradation of phosphorylated SMAD1/5/9, and thereby inhibit BMP pathway signaling to promote neuronal differentiation (Geng et al., 2021). Further studies of this proposed mechanism are needed to evaluate its relevance to cortical neuron differentiation *in vivo*, since the aforementioned studies were done in cultured HEK cells or mESCs, after overexpression of AUTS2 or proposed interacting molecules.

## Neurodevelopmental Functions of AUTS2 in Animal Model Systems

### Zebrafish

The first animal studies of AUTS2 neurodevelopmental functions were conducted in zebrafish, using splice-blocking and translation-blocking morpholinos to knock down *auts2a* (Beunders et al., 2013; Oksenberg et al., 2013). In *auts2a* morphants, AUTS2 deficiency resulted in microcephaly, decreased neurogenesis, and other growth defects (Table 2). These phenotypes were rescued by expression of either AUTS2-FL or AUTS2-C, indicating that important AUTS2 functions are retained in the C-terminal region (Beunders et al., 2013). Interestingly, one study in zebrafish observed micrognathia/retrognathia in *auts2a* morphants, replicating a phenotype observed in humans (Beunders et al., 2013). Jaw growth may potentially be a conserved function of AUTS2, related to the evolutionary amplification of *AUTS2* ohnologs in gnathostomes (Sellers et al., 2020). However, since zebrafish lack laminated cerebral cortex, and morphants often exhibit off-target effects (Vogan, 2015), the utility of zebrafish for studying human brain development is very limited. Moreover, zebrafish have a second gene, *auts2b*, also expressed in developing brain (Kondrychyn et al., 2017).

**TABLE 2:** Animal models of *AUTS2* syndrome.

Species	Perturbation	Phenotypes	Rescue	References
Zebrafish	sb-morpholinos against *auts2a*	Microcephaly. Micrognathia. Retrognathia. Decreased neurogenesis. Decreased proliferation.	AUTS2-FL AUTS2-C	Beunders et al., 2013
Zebrafish	tb- and sb-morpholinos against *auts2a*	Microcephaly. Microphthalmia. Decreased neurogenesis. Increased proliferation. Increased apoptosis. Fewer spinal motoneurons. Fewer spinal sensory neurons. Increased axon branching.	AUTS2-FL	Oksenberg et al., 2013
Mouse	*Auts2^del7/del7^* (*Nes*-Cre)	No brain abnormalities decreased body growth. Impaired righting reflex. Decreased USVs. Impaired negative geotaxis.		Gao et al., 2014
Mouse	*Auts2^del8/del8^* (whole organism)	No brain abnormalities (P0)neonatal lethal. AUTS2-C upregulated.		Hori et al., 2014
Mouse	*Auts2^neo/+^* (∼50% reduced AUTS2-FL and AUTS2-C)	Decreased anxiety. Decreased exploratory behav. Impaired novel object recog. Impaired cued assoc. memory Increased nociceptive resp. Altered acoustic startle		Hori et al., 2015
Mouse	*Auts2^del8/del8^* (*Emx1*-Cre or *CaMKIIa*-CreER*^T2^*) *Auts2^del8/+^*	Increased dendritic spines. Increased mEPSCs. Decreased social interactions. Decreased exploratory behav. Decreased fear of heights. Impaired novel object recog. Increased nociceptive resp. Decreased prepulse inhibition. Altered acoustic startle Decreased USVs		Hori et al., 2020
Mouse	*Auts2^del8/del8^* (*En1*-Cre)	Cerebellum small, malformed. Decreased Purkinje cells. Decreased MB DA neurons. AUTS2-C upregulated. Impaired motor learning. Decreased male USVs.		Yamashiro et al., 2020
Mouse	*Auts2^del15/del15^* (*Nes*-Cre) *Auts2^del15/del15^* (*Emx1*-Cre)	Neonatal lethal. Abnormal breathing rhythms. Dentate gyrus small. Abnormal EEG.		Castanza et al., 2021

### Mice

Several mouse models of AUTS2 deficiency have been produced by gene targeting of different *Auts2* exons (Table 2). The structure of the *Auts2* gene in mice (1.1 Mb, chromosome 5) is similar as in humans, and likewise comprises 19 exons (Castanza et al., 2021). The alternative TSS in exon 9 is active in mice and produces a C-terminal AUTS2 isoform similar to that in humans (Hori et al., 2014). In addition, mice have another alternative TSS in exon 7, which uses a translational start site in exon 8 and produces a slightly longer C-terminal isoform (Hori et al., 2014). Analyses of protein and RNA indicate that AUTS2-FL and AUTS2-C isoforms are both expressed in developing mouse brain, with AUTS2-C isoforms predominating in embryonic cerebral cortex, and AUTS2-FL in cerebellum (Gao et al., 2014; Hori et al., 2014; Molyneaux et al., 2015; Castanza et al., 2021).

In mice lacking *Auts2* exon 7 (*Auts2^del7/del7^*) in the nervous system (*Nes*-Cre), mutants had normal birth weight but grew more slowly than controls postnatally, and exhibited behavioral abnormalities such as decreased ultrasonic vocalizations (Table 2) (Gao et al., 2014). No structural brain defects were reported, and only one gene (*Dynll1*) was significantly dysregulated among a panel of 9 candidate *Auts2* target genes assayed by RT-PCR. Importantly, excision of exon 7 did not disrupt the exon 9 TSS, and AUTS2-C was presumably still expressed, although this was not tested (Gao et al., 2014). Thus, this model likely caused only partial loss of AUTS2 function, related to the full-length isoform.

In another mouse model, mutants lacking *Auts2* exon 8 (*Auts2^del8/del8^*) throughout the organism died in the neonatal period (Table 2) (Hori et al., 2014). The neonatal brains showed no abnormalities by macroscopic examination or histology. In this model, the alternative TSS in exon 9 was again undisturbed, and AUTS2-C protein was actually increased in the cerebral cortex of *Auts2^del8/del8^* mice, possibly as a compensatory response to AUTS2-FL depletion (Hori et al., 2014). Conditional excision of exon 8 in cerebral cortex pyramidal neurons during development (*Emx1*-Cre) or adulthood (*CaMKIIa*-CreERT2) permitted longer postnatal survival and additional studies (Hori et al., 2020). In these models, increased numbers of dendritic spines were observed on pyramidal neurons in the hippocampus (CA1) and neocortical layers 2–3 of mutant mice, along with increased numbers of miniature excitatory postsynaptic currents (mEPSCs), as well as behavioral abnormalities (Hori et al., 2020). Conditional deletion of *Auts2* exon 8 in the developing cerebellum and caudal midbrain (*En1*-Cre) caused cerebellar hypoplasia, with defects of Purkinje cell maturation and synaptogenesis, plus behavioral abnormalities (Yamashiro et al., 2020). The latter results accord with previous evidence that AUTS2-FL is the main isoform in developing cerebellum (Castanza et al., 2021). Importantly, the cerebellar hypoplasia in mice (Yamashiro et al., 2020) recapitulates the cerebellar hypoplasia seen in some AUTS2 syndrome patients with missense or microdeletion variants in exon 9 (Liu et al., 2021; Fair et al., 2022).

In the course of producing *Auts2^del8^*, an unexpected loss-of-function allele (*Auts2^neo^*) that reduces both AUTS2-FL and AUTS2-C was also produced (Table 2) (Hori et al., 2014). Like *Auts2^del8/del8^*, the *Auts2^neo/neo^* genotype caused neonatal lethality. Heterozygous *Auts2^neo/+^* mice, reported to have ∼50% reduction of AUTS2-FL and AUTS2-C isoforms, showed behavioral abnormalities (Hori et al., 2015). Since human AUTS2 mutations are heterozygous (Beunders et al., 2013; Sanchez-Jimeno et al., 2021), *Auts2^neo/+^* mice model the human AUTS2 syndrome in this regard.

Recently, a conditional exon 15 allele (*Auts2^del15^*) was produced to interfere with expression of both AUTS2-FL and AUTS2-C isoforms (Table 2) (Castanza et al., 2021). Mice lacking exon 15 (*Auts2^del15/del15^*) throughout the CNS (*Nes*-Cre) died neonatally with severe breathing abnormalities, documented by plethysmography. The erratic breathing implicated abnormal development of brainstem respiratory centers and raised the possibility that *AUTS2* might be a vulnerability gene for sudden infant death syndrome. Excision of exon 15 only in the developing cortex (*Emx1*-Cre) allowed for survival to adulthood, and revealed defects of cortical structure, function, and gene expression (Castanza et al., 2021). Notably, the dentate gyrus (DG) of hippocampus was hypoplastic, with virtual absence of hilar mossy neurons and reduced numbers of granule neurons. Also, EEG recordings showed abnormal spiking activity. While the neocortex appeared structurally intact, genes involved in neocortical patterning (such as *Tshz2*) and laminar identity (such as *Wnt7b* and *Pcdh20*) were significantly dysregulated, as determined by RNA-seq. Significantly, many RNA transcripts that interacted with AUTS2 in normal neocortex (by RIP-seq) were also dysregulated in *Auts2^del15/del15^* mutant neocortex (by RNA-seq). Indeed, mRNA dysregulation correlated more strongly with binding of the RNA to AUTS2 protein (RIP-seq), than with binding of AUTS2 to cognate genes in chromatin (ChIP-seq). These distinctions support the hypothesis that AUTS2 regulates gene expression mainly by modulating RNA metabolism.

Interestingly, the transcriptome analysis of *Auts2^del15/del15^* neocortex (Castanza et al., 2021) revealed dysregulation of mRNAs for proteins that were previously reported to interact with AUTS2 protein. Specifically, transcripts *Ep300* and *Prex1* not only bound AUTS2, but also were significantly decreased in *Auts2^del15/del15^* neocortex. *Ep300* and *Prex1* encode P300 and P-Rex1, respectively, both of which reportedly interacted with AUTS2 upon overexpression in HEK cells (Gao et al., 2014; Hori et al., 2014). These findings suggest that overexpression of AUTS2 might induce elevated levels of *Ep300* and *Prex1* mRNA, and consequently, likewise elevated levels of P300 and P-Rex1 proteins. Overexpression of P300 and P-Rex1 with AUTS2 might lead to non-physiologic interactions in HEK cells, which are extremely different from neurons in cerebral cortex. Similarly, the activation of Rac1 observed after AUTS2 overexpression in N1E-115 mouse neuroblastoma cells (the “AUTS2-Rac1 pathway”) may also be explained, at least in part, by AUTS2 regulation of transcripts *Prex1* and *Rasgrf2*, which were significantly reduced in *Auts2^del15/del15^* neocortex. Both *Prex1* and *Rasgrf2* encode GEFs that activate Rac1 (Yoshizawa et al., 2005; Li et al., 2006). Thus, AUTS2 overexpression in HEK cells might indirectly induce elevated levels of GEFs via RNA regulation, and thereby activate Rac1.

Finally, a novel mouse *Auts2* allele with conditional excision of exon 9 (*Auts2^del9^*) was recently described (Geng et al., 2021). Excision of exon 9 was reported to deplete both AUTS2-FL and AUTS2-C, although no details of the generation of these mice, nor documentation of the protein depletion, were provided. Homozygous excision of exon 9 (*Auts2^del9/del9^*) in the central nervous system (*Nes*-Cre) was reported to cause early embryonic lethality. Heterozygous exon 9 excision was found to impair neuronal gene expression, as determined by RT-PCR of cultured neocortical cells (Geng et al., 2021). No brain abnormalities were reported. Further studies are needed to validate this model.

## Mouse Embryonic Stem Cell Models

The question of whether AUTS2 regulates neurogenesis has also been studied using mouse embryonic stem cells (mESCs) treated with *in vitro* differentiation protocols. One group reported that AUTS2 fosters the differentiation of mESCs to motor neurons (MNs) (Liu et al., 2021). Interestingly, a reduction of spinal MNs was reported in *auts2a* morphant zebrafish (Oksenberg et al., 2013); however, no MN defects have been reported in human *AUTS2* patients or mouse models. In another study, AUTS2 was found necessary for the differentiation of mESCs to mixed neuron types (Russo et al., 2018). For cortical-type neurons, AUTS2 facilitated the differentiation of mESCs to cortical neurons in one study (Geng et al., 2021), but delayed mESC differentiation to cortical neurons in another (Monderer-Rothkoff et al., 2021). WDR68, implicated as an essential binding partner for transcriptional activation by AUTS2, also appeared necessary for the differentiation of mESCs to cortical neurons (Wang et al., 2018). While defects of neurogenesis presumably cause microcephaly in human AUTS2 syndrome, mESCs have so far shown variable results and limited utility for understanding AUTS2 syndrome phenotypes.

## Cerebral Organoids as a Model to Investigate Molecular Mechanisms Underlying AUTS2 Syndrome

Mouse models of AUTS2 deficiency have furthered our understanding of AUTS2 functions during neurodevelopment. However, the role of AUTS2 during human cortical development, which involves a greater expansion of radial glia cell and neuronal diversity compared to mice, has not been investigated until recently with cerebral organoids (COs) (Fair et al., 2022). COs, derived from human pluripotent stem cells, are three-dimensional *in vitro* models that can be utilized to investigate the complex and human-specific features of early brain development (Lancaster et al., 2013; Di Lullo and Kriegstein, 2017; Pasca, 2018; Pollen et al., 2019; Qian et al., 2019; Setia and Muotri, 2019). As COs have been extensively reviewed elsewhere, we will focus on how COs can be used to investigate the mechanisms of aberrant human corticogenesis underlying various neurodevelopmental disorders (NDs), emphasizing studies relevant to AUTS2 syndrome.

**FIGURE 4:**
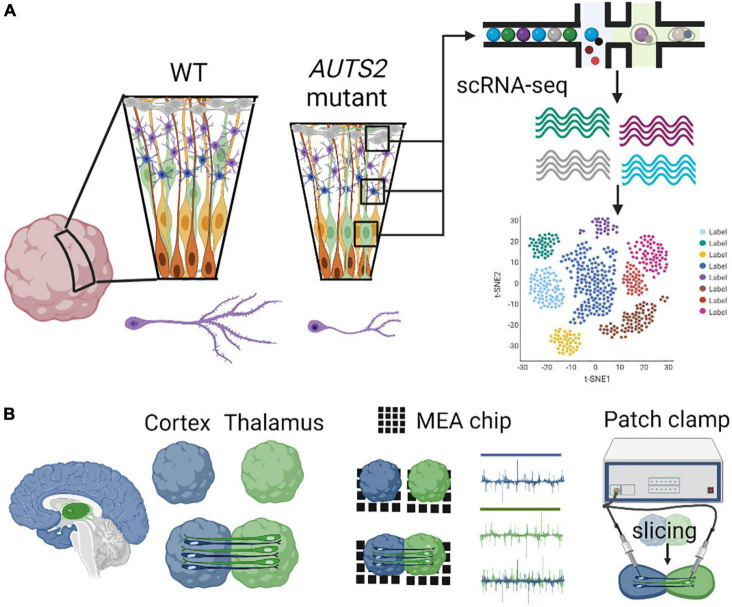
Modeling AUTS2 syndrome using cerebral organoids. **(A)** *AUTS2* mutant cerebral organoids have proliferative deficits in neural progenitor cells and impaired neuritogenesis in cortical neurons (left). Utilization of scRNA-seq to determine cell type specific changes in gene expression within diverse progenitor, neuronal, and glial cell populations (right). **(B)** Generation of assembloids through the fusion of brain region specific organoids (cortical-thalamic assembloid shown, left) investigating neural circuitry using MEA and patch-clamp electrophysiology (right).

Recently, the first COs derived from an AUTS2 syndrome patient, with a pathogenic missense variant in exon 9, were generated and studied (Fair et al., 2022). Patient COs displayed reduced growth analogous to microcephaly, deficits in neural progenitor cell (NPC) proliferation, and abnormal neuronal differentiation, which were rescued by CRISPR-Cas9 gene editing of the variant to the wild-type allele (Figure 4). These data highlight essential roles for AUTS2 during early human cortical development as well as identified proliferative deficits and reduced WNT-β-Catenin signaling in NPCs, which may underlie microcephaly in AUTS2 syndrome. Gene expression signatures of defective neuritogenesis were also observed, similar findings which are consistent with previous mouse studies. Future investigation is necessary to understand how AUTS2 functions in various progenitor cells (e.g., apical, intermediate, and basal), how it regulates neuronal differentiation, and how it controls the formation of neuronal circuits. The development and comparison of additional brain region-specific COs coupled with advanced technological tools to investigate various patient *AUTS2* variants can start to uncover neurobiological functions that contribute to the clinical heterogeneity observed in AUTS2 syndrome patients (Figure 4).

## Investigating Mechanisms Underlying Aberrant Corticogenesis in AUTS2 Deficient Cerebral Organoids

Disruptions in the *AUTS2* gene are associated with microcephaly in human patients (Beunders et al., 2013; Sanchez-Jimeno et al., 2021). However, current mouse models of Auts2 deficiency have not been able to recapitulate a microcephalic phenotype (Gao et al., 2014; Hori et al., 2014; Castanza et al., 2021). Understanding the etiology of microcephaly in AUTS2 and other neurodevelopmental disorders has been limited by the primary use of mouse models which do not adequately recapitulate the manner and severity in which microcephaly arises (Gabriel et al., 2020; Nieto-Estevez and Hsieh, 2020). A major developmental difference occurs during neurogenesis between humans and mice, particularly in the generation of the subventricular zone (SVZ). Although both humans and rodents undergo extensive SVZ growth, humans have a more complex, organized, and expansive SVZ subdivided into the outer and inner SVZ (Borrell and Gotz, 2014; Florio and Huttner, 2014; Florio et al., 2018). This compartmentalization allows for a more heterogeneous population of neural precursor cells with dynamic proliferative capabilities (such as cell cycle length and mode of division). Subsequently, the cortical plate is massively expanded in humans and becomes highly folded (undergoes gyrification) whereas the mouse brain is smooth (lissencephalic) (Kelava and Lancaster, 2016; Gabriel et al., 2020). Thus, major variations exist between human and rodent brain development resulting in dramatic differences in brain composition, size, and complexity.

As a complementary system to mouse models, COs can be used to dissect the underlying mechanisms of microcephaly while also helping to elucidate the fundamental mechanism of normal human brain development (Gopalakrishnan, 2019; Setia and Muotri, 2019; Gabriel et al., 2020). Microcephaly is thought to arise from a common disease mechanism ultimately owing to dysregulation of the cell cycle that disrupts the timing of this carefully orchestrated neurogenesis (Jean et al., 2020). It is hypothesized that human brain development occurs in distinct stages of cell proliferation and subsequent differentiation, whereas mice may undergo both proliferation and differentiation simultaneously (Geschwind and Rakic, 2013; Florio and Huttner, 2014; Gabriel et al., 2020). Furthermore, mouse models of candidate microcephaly genes typically require homozygous inactivation of the causal gene or significant mutant gene overexpression in order to induce a phenotype. However, primary microcephaly in humans is caused predominately by gene point mutations and truncations (Barrera et al., 2010; Buchman et al., 2010; Lizarraga et al., 2010; Alkuraya et al., 2011; McIntyre et al., 2012; Insolera et al., 2014). For example, mouse models of microcephaly associated CDK5RAP2 disease requires a complete knockout of *CDK5RAP2* and result in apoptosis as the primary cause of neuronal progenitor cell depletion and microcephaly (Barrera et al., 2010; Buchman et al., 2010; Lizarraga et al., 2010). Conversely, a patient-derived CO model containing a heterozygous truncating mutation in the *CDK5RAP2* gene recapitulated severe microcephaly and resulted in fewer neurons and smaller progenitor zones (Lancaster et al., 2013). Further investigation of radial glial spindle orientations revealed that patient COs displayed a greater percentage of oblique and vertical divisions compared to controls. This observation in patient COs correlated with premature neural differentiation, which was further supported by increased BrdU+/doublecortin positive cells.

Similarly, a recent study modeling a pathogenic *AUTS2* missense variant in COs revealed significantly reduced organoid growth (Fair et al., 2022). AUTS2 mutant COs showed dysregulated cell cycle control and reduced symmetrical (horizontal) cellular division, which correlated with premature neuronal differentiation in comparison to control COs. Increased asymmetrical progenitor divisions and premature neuronal differentiation are also common features observed in other CO models containing mutant microcephaly genes (Gabriel et al., 2020). These data support the role of AUTS2 in regulating the highly intricate transcriptional program of neuronal differentiation and perturbations in this process could underlie cognitive deficits in patients.

Although mice do not have an OSVZ, both OSVZ formation and DG morphogenesis involve extensive cell migration and proliferation (Molnár et al., 2019; Nelson et al., 2020). Castanza et al. (2021) used a conditional knock-out model to inactivate *Auts2* specifically in the cerebral cortex and found that the dentate gyrus had a reduced size that was associated with decreased neurogenesis, a common feature of AUTS2-associated microcephaly. It is possible that similar molecular mechanisms that perturbs DG neurogenesis in AUTS2 deficient mice, may contribute to neocortical growth defects in human AUTS2 syndrome patients.

## Genomics Technologies to Study Molecular Features of AUTS2 Deficiency in Cerebral Organoids

Single cell (sc) RNA-seq, combined with immunohistochemical, spatial transcriptomics, and chromatin immunoprecipitation techniques can provide important morphological context to reconstruct the organization of disease-related expression patterns (Rao et al., 2021). Early rosette structures in COs represent ventricular zone (VZ)-like structures that are comprised of stratified progenitors, which undergo distinct stromal translocations critical to their progression through the cell cycle. Apical neural progenitors divide symmetrically within VZ-like structures in COs, and at the onset of neurogenesis, they shift to asymmetric divisions forming another apical neural progenitor and either a neuron or an intermediate progenitor (IP) cell, which is a type of basal neural progenitor. Although apical neural progenitors can directly give rise to neurons, most neurogenesis in the human neocortex occurs indirectly through subsequent IP division and neural differentiation (Pebworth et al., 2021). In contrast to rodents, humans and larger mammals contain an abundant type of basal progenitor called outer radial glial (ORG) cells, which also give rise to IP cells and are formed in the OSVZ. COs are invaluable models of human brain development since they contain an outer subventricular zone (oSVZ), which are abundant in ORG (Kadoshima et al., 2013; Lancaster et al., 2013) (Figure 4).

Recent work identified altered morphological and functional properties of apical neural progenitors in AUTS2 deficient COs (Fair et al., 2022). Further investigation of AUTS2 function using immunofluorescence techniques and sc-RNA seq will provide deeper understanding for the role of AUTS in IP and ORG populations in human corticogenesis and in AUTS2 syndrome. Correlating spatial progenitor zone context with cell type specific transcriptomic signatures may provide further mechanistic insight into whether downstream targets of AUTS2 are affected in specific progenitor zone populations in COs. Additionally, combining sc and epigenetic sequencing methods may also provide critical insights into altered regions of chromatin accessibility leading to deficits in transcriptional control caused by AUTS2 deficiency.

Although ChIP-seq is a widely used method to evaluate global active and repressed chromatin states, it is limited by low signal-to-noise ratios and is not compatible with low cellular input–a potentially significant challenge for organoid applications (Lewis et al., 2021). However, newer chromatin mapping methods such as CUT&RUN and CUT&Tag can be combined with scRNA-seq to target and profile specific chromatin signatures with single cell resolution (Kaya-Okur et al., 2019; Yu et al., 2022). Single-cell chromatin profiling of AUTS2 syndrome organoid models could extend our understanding for the role of the PRC1-AUTS2 complex in epigenetically heterogeneous cell populations. Such studies could also be valuable to understand the function of *AUTS2* variants in regard to cell-type specific regulatory elements, especially those related to cell cycle control, cell fate inheritance, and timing of neuronal differentiation.

Recently, sc-RNA seq and ATAC-seq analyses uncovered a dynamic period of chromatin remodeling during the development of human forebrain organoids (Trevino et al., 2020). Dynamic epigenetic changes were identified within these organoids during human cortical neurogenesis, driven by specific transcription factors (particularly those associated with astrocyte maturation and interneuron specification). Furthermore, direct comparisons with human tissue confirmed that *in vivo* forebrain regulatory programs largely map with those seen in forebrain organoids cultured for over 20 months. Interestingly, during forebrain organoid development, 81% of Simons Foundation Autism Research Initiative (SFARI) genes were found to be expressed (Trevino et al., 2020). Further analysis identified SFARI genes as significantly enriched for enhancer-gene linkages predominantly in glial progenitor and mature neural cell types. These data provide a foundation for future investigation of potential disease mechanisms in ASD and hold implications for understanding molecular and cellular mechanisms underlying AUTS2 syndrome.

## Electrophysiological Tools to Investigate the Role of AUTS2 in Synaptic Transmission

Several studies have shown that loss of *Auts2* results in dysregulated regional and laminar neuronal differentiation in mouse models (Hori et al., 2020; Castanza et al., 2021; Monderer-Rothkoff et al., 2021) and more recently in COs (Fair et al., 2022). However, the neurodevelopmental impact of altered differentiation on synaptic physiology and how loss of AUTS2 leads to epilepsy in AUTS2 syndrome remains unclear. One study showed that the elimination of dendritic spines was impaired in *Auts2*-knocked-down hippocampal neurons (Hori et al., 2020). As a result, an excess of dendritic spines was observed and was suggested to drive the increase of excitatory inputs within Auts2 mutant hippocampal slices as demonstrated through electrophysiological experiments (Hori et al., 2020). Further studies revealed alterations in synaptic gene regulation in both *Auts2* mutant mouse hippocampus and frontal cortex (Hori et al., 2020; Castanza et al., 2021), and in a CO model containing an *AUTS2* pathogenic variant (Fair et al., 2022). All of these studies suggest AUTS2 functions at excitatory synapses, however, the mechanisms of AUTS2 in establishing synaptic maturation and regulating synaptic homeostasis during neurodevelopment remains largely unknown.

To investigate synaptic functions *in vitro*, conventional patch-clamp recording techniques remain the gold standard for providing high temporal resolution of electrical activities in neurons. Whole-cell voltage-clamp recordings on acute hippocampal slices from conditional AUTS2 deficient (*Auts2^del8/del8^*; *Emx1*-Cre) mice (P33–P44) revealed an increased frequency of miniature excitatory postsynaptic currents (mEPSCs) with no change in amplitude, suggesting an increase of functional excitatory synapses (Table 2). Further investigations at earlier developmental time points and across different regions in Auts2 deficient brains would provide a more complete understanding of Auts2 function in synaptic transmission.

Although patch-clamp techniques offer multiple advantages to probe synaptic function in neuronal cultures, its limited throughput nature does not allow for probing neuronal connectivity and investigating neural network dynamics. To address this limitation, multi-electrode array (MEA) platforms have been extensively utilized to record extracellular field potentials from a diverse and large number of neurons *in vitro* to investigate neural network properties. The application of MEAs in studying the effects of AUTS2 deficiency on neuronal network dynamics has not been explored, and thus would provide further mechanistic understanding of AUTS2 function in synaptic transmission (Figure 4).

Several studies have investigated electrical properties and neuronal connectivity within CO models (Quadrato et al., 2017; Trujillo et al., 2019; Fair et al., 2020). MEAs provide the unique advantage of non-invasively measuring electrical activity of neuronal networks within developing COs, although their utilization to study mechanisms underlie ASD is underexplored. Thus, investigation of spontaneous electrical activities and potential alterations in neural network properties in AUTS2 deficient COs would lead to greater understanding of mechanisms underlying epilepsy in AUTS2 syndrome.

## Generation of Brain Region-Specific Cerebral Organoids and Assembloids to Understand the Role of AUTS2 During Neurodevelopment

Animal studies have shown that *Auts2* is dynamically regulated during brain development and is expressed from a gradient in a high rostral to low caudal pattern within the developing neocortex (Bedogni et al., 2010). As previously described, two main AUTS2 isoforms are differentially regulated during brain development, although the regulatory mechanisms and functions of these isoforms are not well established (Table 1). Owing to this complex and heterogenous expression profile, it is important to understand the precise role of these isoforms within different brain regions throughout development. Numerous protocols exist to generate either brain region specific or whole brain organoids. In this section, we will discuss application of brain region specific organoid and assembloid models that can be leveraged to investigate Auts2 function during neurodevelopment.

Postnatal AUTS2 expression includes high expression levels frontal cortex, cerebellum, and hippocampus (Figure 2). However, it is unclear whether postnatal AUTS2 expression is essential to maintain proper function within these brain regions (Table 1). One hypothesis is that AUTS2 deficiency within the developing hippocampus may underlie ID in AUTS2 syndrome patients. Hippocampal organoids serve as *in vitro* models for investigating human hippocampus development and hippocampus-related diseases and consist of hippocampal granule- and pyramidal-like neurons that form a functional electrical network (Sakaguchi et al., 2015; Pomeshchik et al., 2020). Recently used to model Alzheimer’s disease (Sakaguchi et al., 2015; Pomeshchik et al., 2020), hippocampal organoids can also be applied to understand the role of AUTS2 during hippocampus development. As previously described, Castanza et al. (2021) showed dentate gyrus hypoplasia in *Emx1*-Cre *Auts2^del15/del15^* mice. Utilization of hippocampal organoids can yield important mechanistic insights into the role of AUTS2 in progenitor proliferation, migration, and differentiation within human hippocampal development.

Cerebellar organoids can also be leveraged to understand the role of AUTS2 during cerebellar development. Mouse models of AUTS2 deficiency show cerebellar hypoplasia, impaired dendrite maturation in Purkinje cells, increased parallel fiber synapse formation, and decreased number of excitatory presynaptic synapses from climbing fiber innervations (Yamashiro et al., 2020). Previous work utilizing a polarized cerebellar organoid model identified similarities in human cerebellar ontogenesis regarding the layered neural-tube-like structure with dorsoventral and apicobasal polarities (Muguruma et al., 2015; Silva et al., 2020; Nayler et al., 2021). Cerebellar organoids derived from AUTS2 syndrome patients could be generated to further understand multiple aspects of cerebellar development, such as cellular differentiation, gene expression changes within specific cell types (i.e., sc RNA-seq focusing on Purkinje cells, granular cells, etc.), and investigating E/I balance in the cerebellum utilizing MEA or patch clamp electrophysiology (Figure 4).

Though the development of *in vitro* tools to model and study long-range human brain circuits has remained a challenging endeavor, novel platforms are emerging based on the fusion of regionally-specified brain organoids called assembloids. For instance, the neural circuitry between the human thalamus and cerebral cortex can be mimicked and studied in a cortico-thalamic assembloid (Gobbo et al., 2021) (Figure 4). Assembloids are becoming increasingly important, serving as a functional tool to investigate the underlying causes of synaptic deficits underlying neurodevelopmental disorders. A variety of assembloids are being developed to investigate multi-synaptic circuitry in human models, such as cortico-thalamic, cortico-striatal, cortico-cortico assembloids (Xiang et al., 2019; Andersen et al., 2020; Chen et al., 2020; Marton and Pasca, 2020). Cortico-thalamic and cortico-striatal dysfunction has been associated with neurodevelopmental disorders and associated co-morbidities such as epilepsy (Shepherd, 2013; He et al., 2021). AUTS2 is highly expressed in the dorsal thalamus and in the striatum (Bedogni et al., 2010), to study neural circuitry between the frontal cortex and these brain regions. To investigate the role of AUTS2 within the prefrontal-thalamic circuit, an assembloid model could be recapitulated between forebrain and thalamic organoids, and several methodologies such as patch clamping or MEAs could be used to investigate alterations in synaptic transmission occurring in assembloid circuits (Figure 4).

The utilization of patient *AUTS2* variant iPSCs to create assembloids and brain region specific organoids, such as hippocampal and cerebellar organoids, will provide invaluable tools to understand mechanisms underlying AUTS2 syndrome at multiple levels of analysis including molecular, cellular, and functional levels, as well as provide a novel screening platform for developing therapeutics.

## Molecular Tools for Analyzing the Role of AUTS2 in Neural Circuit Development and Maintenance

*Auts2* conditional knockout mouse models are invaluable for dissecting the circuit mechanisms that may contribute to phenotypes observed in AUTS2 syndrome. As noted above, mouse models for Auts2 deficiency do not demonstrate microcephaly (Castanza et al., 2021). However, loss of *Auts2* causes changes in molecular expression that suggest disrupted neuronal differentiation, which should lead to altered circuit architecture (Castanza et al., 2021; Monderer-Rothkoff et al., 2021). Indeed, in some models, the density of dendritic spines on excitatory neocortical and hippocampal pyramidal cells is abnormally high, with a concomitant increase in the frequency of miniature excitatory postsynaptic currents (mEPSCs) (Hori et al., 2020). This suggests there may be an imbalance in the ratio of excitation to inhibition (E/I) within neural circuits of *Auts2* mouse models. Congruent with this hypothesis, abnormal voltage spikes similar to epileptic interictal discharges occur spontaneously in the hippocampi of *Auts2* cKO mice (Castanza et al., 2021). Disruptions in E/I balance are commonly observed across several animal models of neurodevelopmental disorders (Sohal and Rubenstein, 2019). However, it is important to determine how loss of *Auts2* alters the developmental trajectory of specific neuronal subtypes and circuits, to guide precision therapeutic approaches.

In mouse neocortex, AUTS2 is largely restricted to excitatory projection neurons (Castanza et al., 2021). These cells can be parsed into three major subclasses based on their long-range axonal targets: (1) intratelencephalic (IT) that project across the corpus callosum, (2) pyramidal tract (PT) that project to subcortical structures, and (3) corticothalamic (CT) that project to thalamic nuclei (Harris and Shepherd, 2015). In mice, these three subclasses form unique local circuits among each other and with neighboring inhibitory interneurons (Lee S.H. et al., 2014; Harris and Shepherd, 2015; Wester et al., 2019). Thus, it is important to determine if specific subtypes of projection neurons are uniquely susceptible to loss of *Auts2*.

Based on molecular expression data, current evidence suggests AUTS2 may be involved in development of all 3 cortical projection neuron types. First, AUTS2 is strongly expressed in superficial cortical layers (Bedogni et al., 2010; Castanza et al., 2021), which are populated exclusively by IT-type cells. Second, AUTS2 is also expressed in layer 5, which contains PT-type neurons (Castanza et al., 2021). Third, *Auts2* expression is activated by Tbr1 (Bedogni et al., 2010), which is a transcription factor that promotes the differentiation of IT-, CT-, and PT-type neurons (Hevner et al., 2002; Han et al., 2011; McKenna et al., 2011; Srinivasan et al., 2012). Fourth, AUTS2 can also be co-expressed with CTIP2 (Bedogni et al., 2010), which is a transcription factor prominently associated with PT-type cells. However, a subset of IT-type neurons does express CTIP2 (Harb et al., 2016), which may account for this observation.

Current tools in mice can directly test how loss of *Auts2* differentially alters neocortical circuits involving IT-, CT-, and PT-type neurons. The *Emx1*-Cre mouse line expresses Cre recombinase in progenitors of neocortical and hippocampal projection neurons as early as embryonic day (E) 10.5 (Gorski et al., 2002) and is a powerful strategy to eliminate *Auts2* expression from the forebrain when crossed to *Auts2* floxed mice (Castanza et al., 2021). On this genetic background, IT-, CT-, and PT-type cells can be identified by injecting retrograde tracers or viral vectors into non-overlapping target structures. Using this strategy, it will be possible to determine which subtypes receive abnormally high excitatory synaptic input, and if it originates from neighboring projection neurons in local circuits or from long-range afferents outside of cortex, such as thalamus. Furthermore, *Auts2* can be conditionally deleted from specific subclasses. For example, to target CT-type cells, the *Ntsr1*-Cre line can be used to express Cre in these neurons as early as embryonic day 16.5 (Gong et al., 2007). Superficial IT-type neurons can be targeted by *in utero* electroporation of a plasmid encoding Cre into *Auts2* floxed mice after E14.5. Several tools exist to investigate if loss of *Auts2* from projection neurons alters their synaptic connectivity with neighboring inhibitory interneurons. For example, conditional *Auts2* knockout mice can be crossed to those expressing fluorescent reporters in specific interneuron subtypes (e.g., the *PV*-tdTomato mouse line (Kaiser et al., 2016). Alternatively, AAV vectors are available that encode fluorophores and channelrhodopsin (ChR2) under the control of an inhibitory interneuron-specific promoter [e.g., AAV-m*Dlx*-NLS-mRuby (Chan et al., 2017)].

In the hippocampus, AUTS2 is expressed throughout the canonical tri-synaptic circuit that includes the entorhinal cortex, dentate gyrus, CA3, and CA1 (Bedogni et al., 2010; Castanza et al., 2021). Thus, loss of *Auts2* may disrupt information processing at multiple stages within this structure. In the dentate gyrus, AUTS2 is expressed in two different populations of excitatory projection neurons: granule cells (GCs) and hilar mossy neurons (HMNs). Importantly, forebrain knockout of *Auts2* leads to a significant reduction in the number of HMNs (Castanza et al., 2021). HMNs project within the dentate gyrus both ipsilaterally and contralaterally to regulate the output of GCs, which in turn target region CA3 (Scharfman and Myers, 2016; Danielson et al., 2017). Although HMNs provide excitatory synaptic input to CGs, their primary role may be to disynaptically inhibit GCs via their input to a diverse set of inhibitory interneurons (Jinde et al., 2012; Scharfman and Myers, 2016). Indeed, loss of HMNs is common in temporal lobe epilepsy (Bui et al., 2018) and may be responsible for the interictal-like events observed by Castanza et al. (2021). Several tools are available to study how loss of *Auts2* alters circuits in the dentate gyrus, perhaps leading to disinhibition and hyperexcitability. HMNs can be selectively targeted for expression of fluorophores and ChR2 by injection of retrograde viral vectors into the contralateral dentate gyrus (Danielson et al., 2017). Furthermore, as described above, local inhibitory interneurons can also be selectively targeted using transgenic and viral strategies. Combining these tools should allow the investigation of synaptic connections among HMNs, GCs, and interneurons on the background of conditional forebrain knockout of *Auts2*. An alternative strategy to selectively target HMNs is to use *Crlr*-Cre or *Drd2*-Cre mouse lines (Gangarossa et al., 2012; Jinde et al., 2012), however, the temporal expression patterns of Cre are not well characterized and may not allow for conditional knockout of *Auts2* during early developmental periods. Finally, loss of *Auts2* may lead to hippocampal hyperexcitability via increases in excitatory synapse formation onto CA1 projection neurons (Hori et al., 2020). The laminar structure of the hippocampus should allow future studies to determine if increased excitatory drive to CA1 originates primarily from CA3 or extrahippocampal inputs (entorhinal cortex or thalamus). This can be accomplished by stimulating axonal projections traveling through either the stratum radiatum or the stratum lacunosum moleculare, respectively.

Finally, mouse models offer an opportunity to investigate if communication among different brain structures is affected by loss of *Auts2*. Indeed, several brain structures that prominently express AUTS2 directly target each other for synaptic connections. These include the prefrontal cortex (PFC), ventrolateral/ventromedial (VL/VM) thalamic nuclei, cerebellum, and hippocampus (Bedogni et al., 2010; Castanza et al., 2021). For example, deep cerebellar nuclei project to directly to VL/VM thalamus (Gornati et al., 2018), which in turn projects to the PFC (Collins et al., 2018). Furthermore, CT- and PT-type projection neurons in PFC provide feedback synaptic input to VM thalamus (Collins et al., 2018). Finally, excitatory hippocampal CA1 projection neurons directly target the PFC (Lee A.T. et al., 2014). An important open question is whether loss of *Auts2* alters the afferent input strength of these connections. This can be addressed by injecting AAVs encoding ChR2 into each of these structures to assess synaptic connectivity and physiology via optogenetic assisted circuit mapping (Petreanu et al., 2007; Collins et al., 2018).

## Future Directions and Key Questions

As this review has illustrated, multiple functions have been proposed for AUTS2 (Figure 3) on the basis of studies in multiple model systems (Table 2). Other members of the *AUTS2* gene family (*FBRSL1, FBRS*) and superfamily (such as *tay*) likewise appear to have overlapping, but diversified functions. Importantly, many AUTS2 functions and interactions with other proteins remain to be confirmed, or have been studied in heterologous cell types (such as HEK cells). Considering these caveats, a paramount future goal will be to better define the functions and binding partners of AUTS2 in neurons from cerebral organoids. With several lines of evidence suggesting that AUTS2 is an RNA-binding protein, this possibility can be investigated using methods such as eCLIP. With the advent of methods to produce human COs and assembloids, future studies can go beyond animal models to evaluate AUTS2 molecular functions, binding partners, and neurodevelopmental roles in developing human neural tissues.

## Author Contributions

RH and AB produced figures and supplementary figures. All authors wrote and edited the manuscript.

## Conflict of Interest

The authors declare that the research was conducted in the absence of any commercial or financial relationships that could be construed as a potential conflict of interest.

## Publisher’s Note

All claims expressed in this article are solely those of the authors and do not necessarily represent those of their affiliated organizations, or those of the publisher, the editors and the reviewers. Any product that may be evaluated in this article, or claim that may be made by its manufacturer, is not guaranteed or endorsed by the publisher.

## Footnotes

1
https://genome.ucsc.edu

2
https://robetta.bakerlab.org

3
https://gene-atlas.brainminds.riken.jp/

## Supplementary Material

Supplementary Figure 1 *FBRSL1* transcript and protein. **(A)** The full-length FBRSL1 transcript has 17 exons. **(B)** Protein source exons are indicated in black (odd) and blue (even). Green boxes enclose splice junctions in which amino acids were encoded across the junctions, comprising exon blocs. Red asterisks indicate source exons that encoded a non-integer number of amino acids. Features mapped from protein sequence: ExB, exon bloc; NLS, nuclear localization sequence; IDR, intrinsically disordered region; PRR, Pro-rich repeat; HRR, His-rich repeat; HX, HX repeat; RRR, Arg-rich repeat; CDD, conserved domain database; PD, ProDom predicted domains (domain names in bold also identified in AUTS2); AFsh, AUTS2-FBRSL1 short homology region. **(C)** FBRSL has a high content of IDRs (zigzag lines) and regions enriched in amino acids Pro, His, or Arg (red lines).

Supplementary Figure 2 Predictive modeling of AUTS2 (1–1,199) secondary and tertiary structure. The AUTS2 protein sequence (aa 1–1,199) was modeled using RoseTTAfold. (The C-terminal aa 1,200–1,259 were omitted because only sequences up to 1,199 aa can be analyzed). Left panels show predicted structures, and right panels show error estimates for each aa position, for models 1–5. Overall confidence was low (0.19 on scale 0–1), most likely because no structures of similar sequences were available. All of the models show multiple long stretches of disordered secondary structure (most obvious in model 5), with intervening alpha helices. Models not shown to the same scale.

Supplementary Figure 3 Predictive modeling of FBRSL1 secondary and tertiary structure. The complete FBRSL1 protein sequence (aa 1–1,045) was modeled using RoseTTAfold. Left panels show predicted structures, and right panels show error estimates for each aa position, for models 1–5. Overall confidence was low (0.17 on scale 0–1), most likely because no structures of similar sequences were available. All of the models show multiple long stretches of disordered secondary structure (most obvious in model 4), with intervening alpha helices. Models not shown to the same scale.

Supplementary Figure 4 Predictive modeling of the AUTS2 domain of AUTS2 protein. The AUTS2 domain of AUTS2 protein (aa 645–844) was modeled using RoseTTAfold. Left panels show predicted structures, and right panels show error estimates for each aa position, for models 1–5. Overall confidence was low (0.45 on scale 0–1), most likely because no structures of similar sequences were available. All of the models show stretches of disordered secondary structure with intervening alpha helices. Models not shown to the same scale.

Supplementary Figure 5 Predictive modeling of the AUTS2 domain of FBRSL1 protein. The AUTS2 domain of FBRSL1 protein (aa 587–784) was modeled using RoseTTAfold. Left panels show predicted structures, and right panels show error estimates for each aa position, for models 1–5. Overall confidence was low (0.42 on scale 0–1), most likely because no structures of similar sequences were available. All of the models show stretches of disordered secondary structure with intervening alpha helices. Models not shown to the same scale.

Supplementary Table 1 *AUTS2* cDNA and protein features. Human *AUTS2* cDNA (CCDS5539.1) and AUTS2 protein (NP_056385.1) features.

Supplementary Table 2 *FBRSL1* cDNA and protein features. Human *FBRSL1* cDNA (CCDS45010.1) and FBRSL1 protein (NP_001136113.1) features.

Supplementary Table 3 Comparison of *AUTS2* and *FBRSL1* cDNA and protein features. Exon homologies, amino acid sequence homologies, and contents of IDRs and LCSs.
