# Supplementary figures and images for: AUTS2 Syndrome: Molecular Mechanisms and Model Systems
Source: Front Mol Neurosci. 2022 Mar 31;15:858582. doi: 10.3389/fnmol.2022.858582 (PMC9008325; doi:10.3389/fnmol.2022.858582)

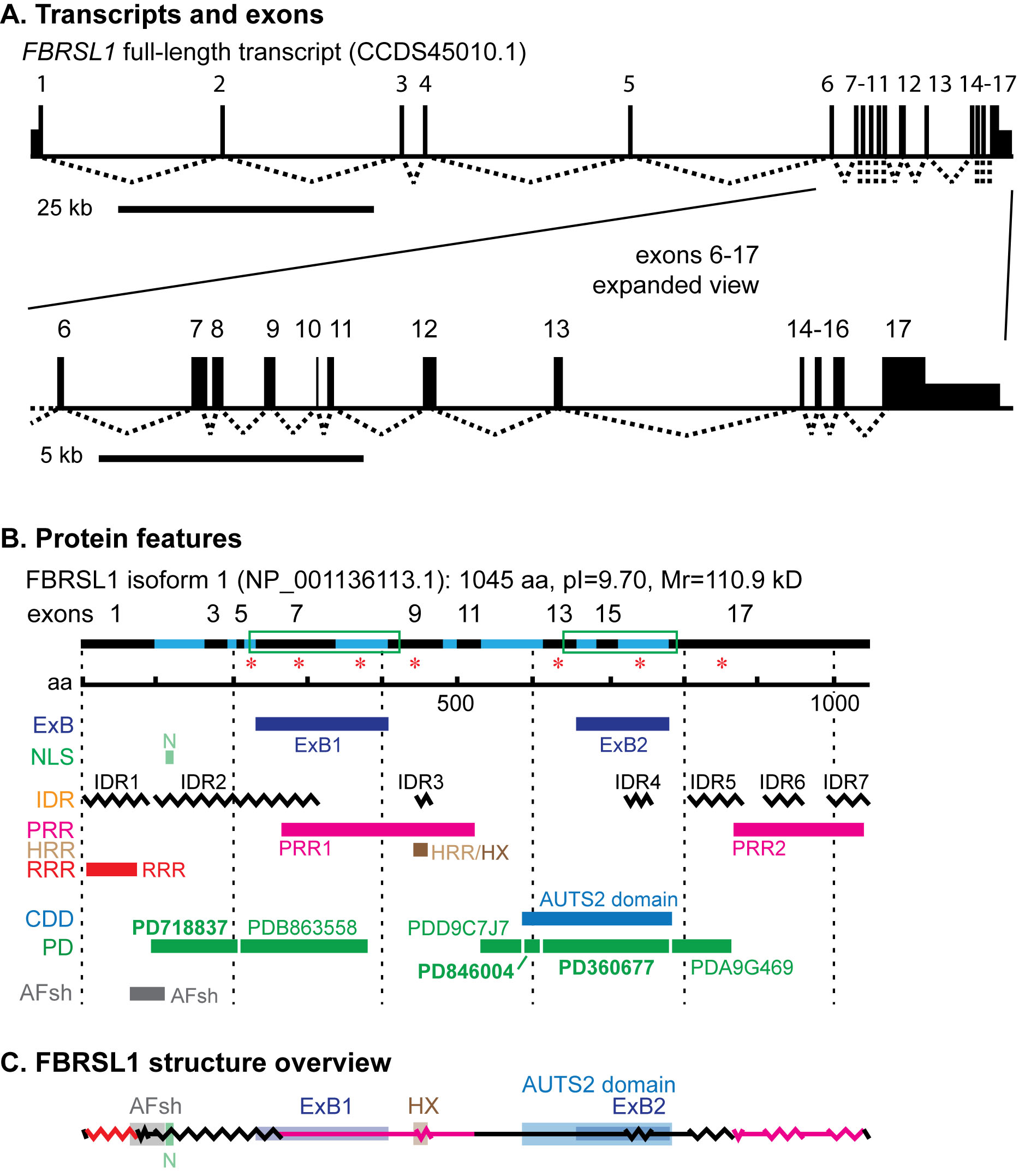

Supplement: Supplementary Figure 1 — FBRSL1 transcript and protein. (A) The full-length FBRSL1 transcript has 17 exons. (B) Protein source exons are indicated in black (odd) and blue (even). Green boxes enclose splice junctions in which amino acids were encoded across the junctions, comprising exon blocs. Red asterisks indicate source exons that encoded a non-integer number of amino acids. Features mapped from protein sequence: ExB, exon bloc; NLS, nuclear localization sequence; IDR, intrinsically disordered region; PRR, Pro-rich repeat; HRR, His-rich repeat; HX, HX repeat; RRR, Arg-rich repeat; CDD, conserved domain database; PD, ProDom predicted domains (domain names in bold also identified in AUTS2); AFsh, AUTS2-FBRSL1 short homology region. (C) FBRSL has a high content of IDRs (zigzag lines) and regions enriched in amino acids Pro, His, or Arg (red lines). [file Image_1.JPEG]

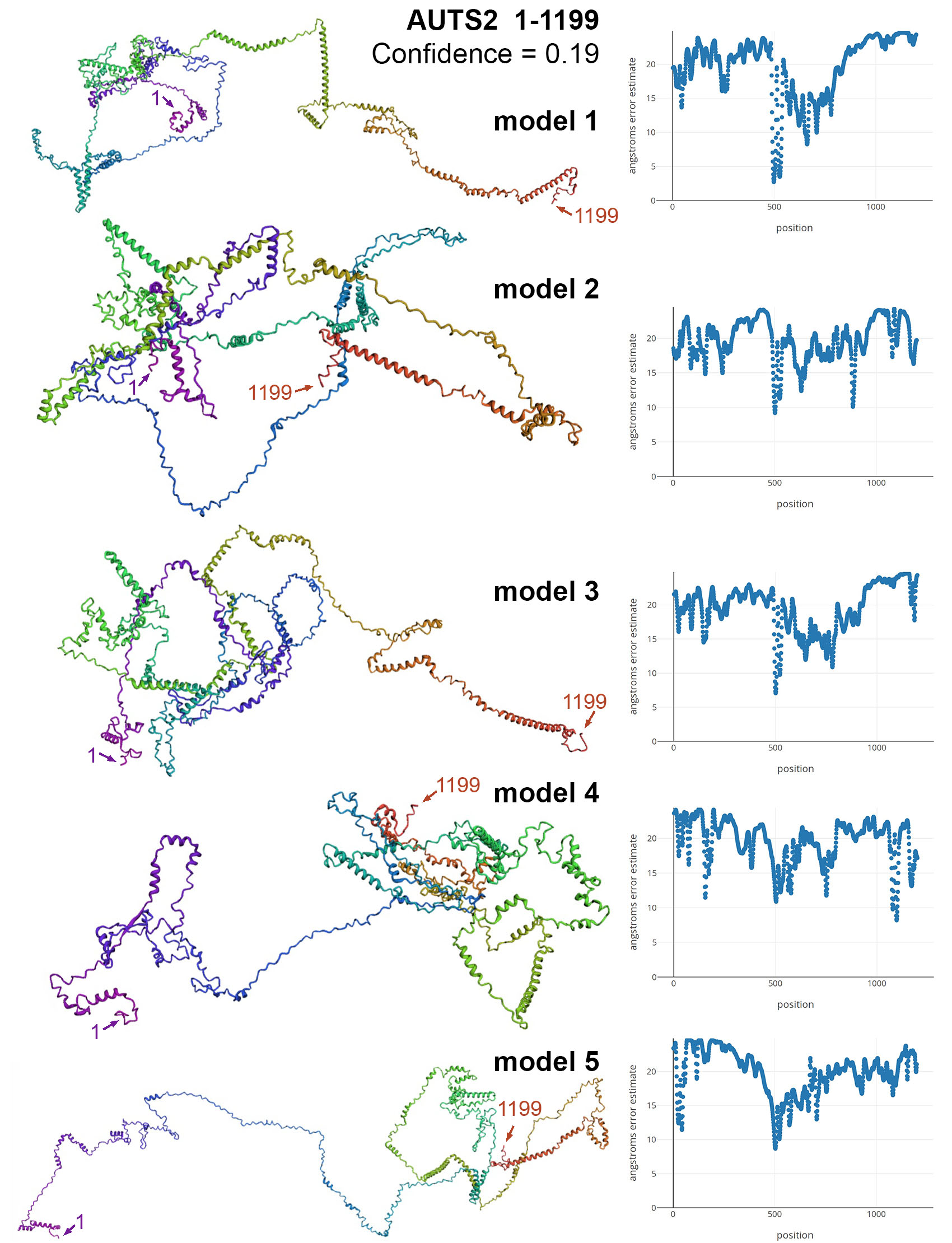

Supplement: Supplementary Figure 2 — Predictive modeling of AUTS2 (1–1,199) secondary and tertiary structure. The AUTS2 protein sequence (aa 1–1,199) was modeled using RoseTTAfold. (The C-terminal aa 1,200–1,259 were omitted because only sequences up to 1,199 aa can be analyzed). Left panels show predicted structures, and right panels show error estimates for each aa position, for models 1–5. Overall confidence was low (0.19 on scale 0–1), most likely because no structures of similar sequences were available. All of the models show multiple long stretches of disordered secondary structure (most obvious in model 5), with intervening alpha helices. Models not shown to the same scale. [file Image_2.JPEG]

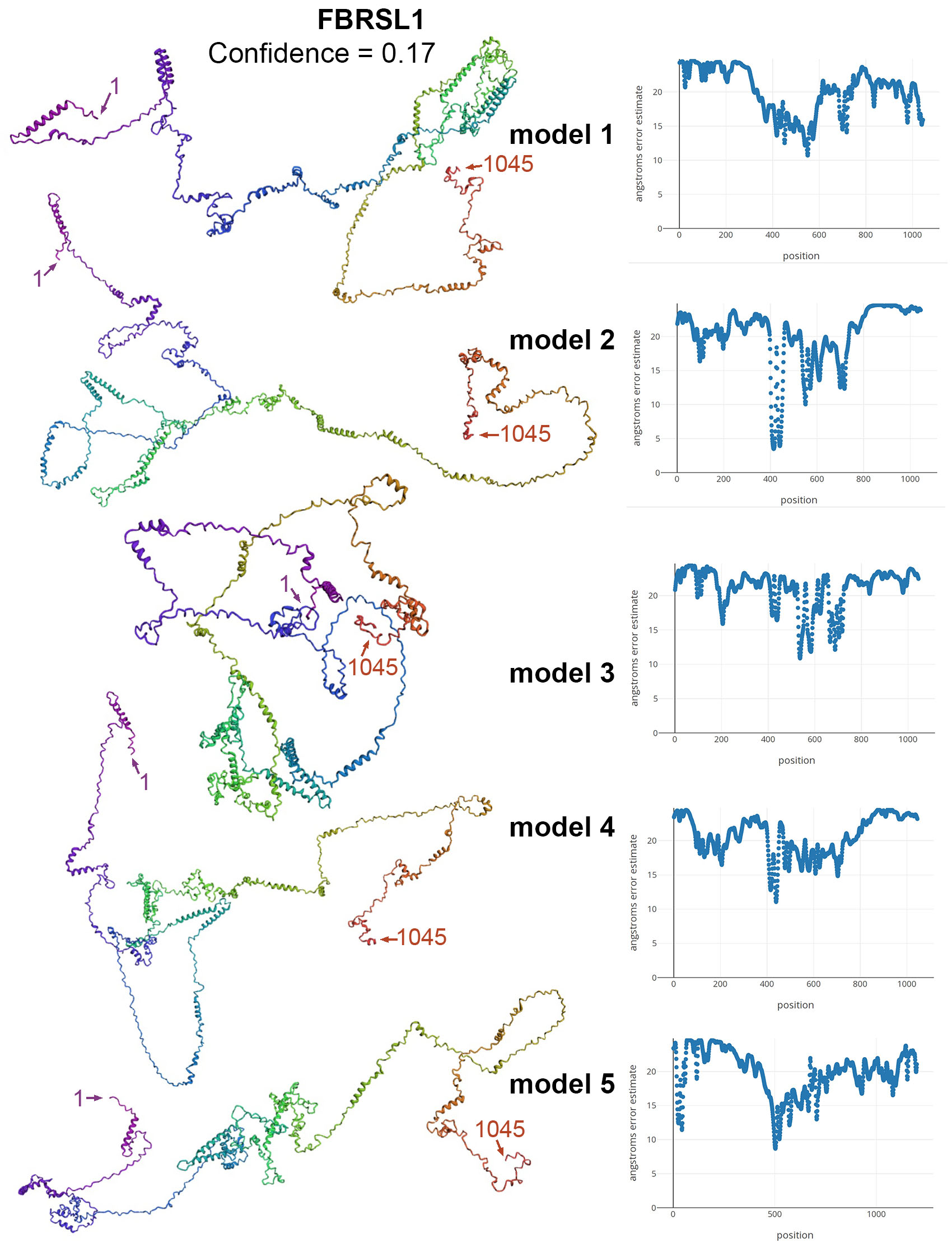

Supplement: Supplementary Figure 3 — Predictive modeling of FBRSL1 secondary and tertiary structure. The complete FBRSL1 protein sequence (aa 1–1,045) was modeled using RoseTTAfold. Left panels show predicted structures, and right panels show error estimates for each aa position, for models 1–5. Overall confidence was low (0.17 on scale 0–1), most likely because no structures of similar sequences were available. All of the models show multiple long stretches of disordered secondary structure (most obvious in model 4), with intervening alpha helices. Models not shown to the same scale. [file Image_3.JPEG]

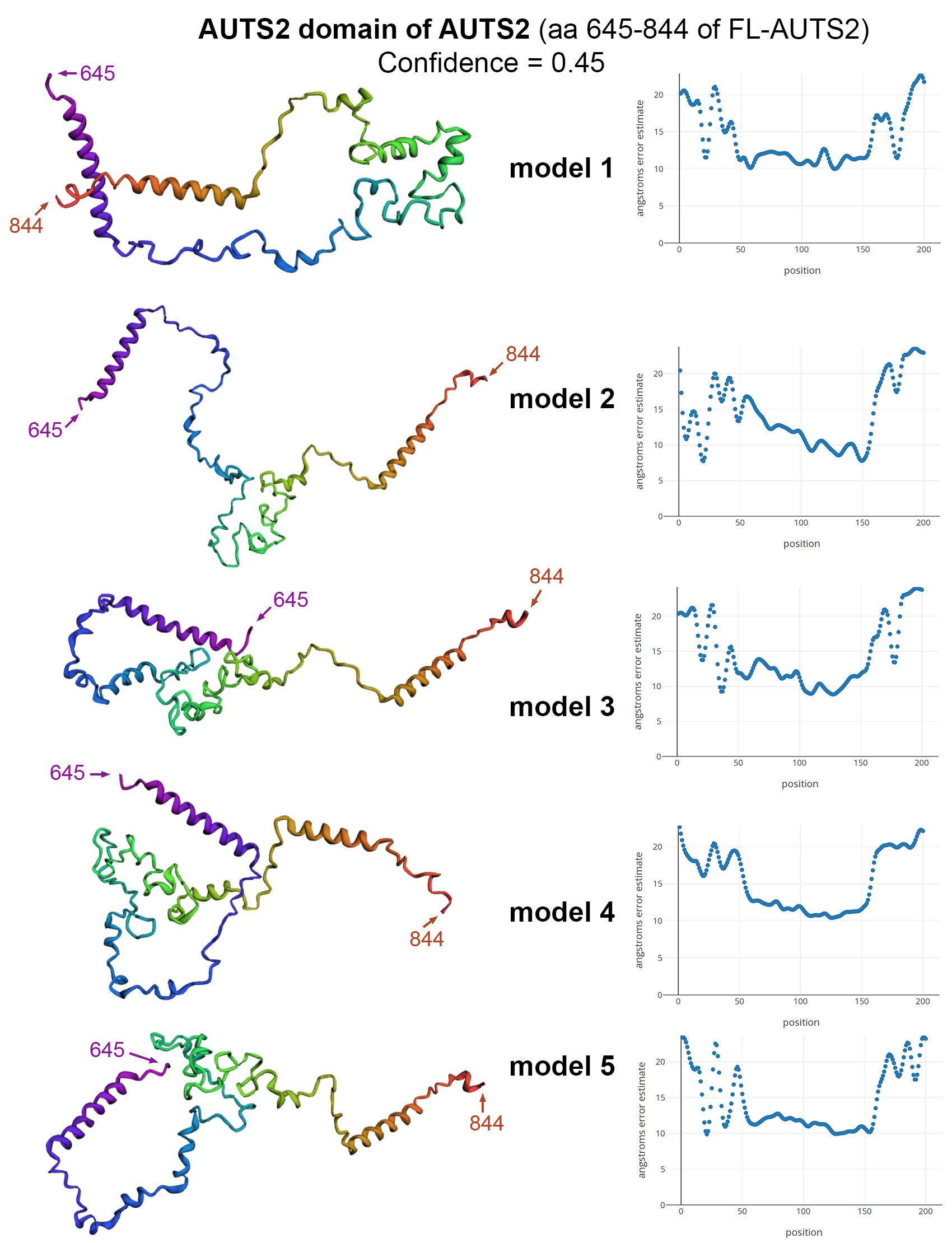

Supplement: Supplementary Figure 4 — Predictive modeling of the AUTS2 domain of AUTS2 protein. The AUTS2 domain of AUTS2 protein (aa 645–844) was modeled using RoseTTAfold. Left panels show predicted structures, and right panels show error estimates for each aa position, for models 1–5. Overall confidence was low (0.45 on scale 0–1), most likely because no structures of similar sequences were available. All of the models show stretches of disordered secondary structure with intervening alpha helices. Models not shown to the same scale. [file Image_4.JPEG]

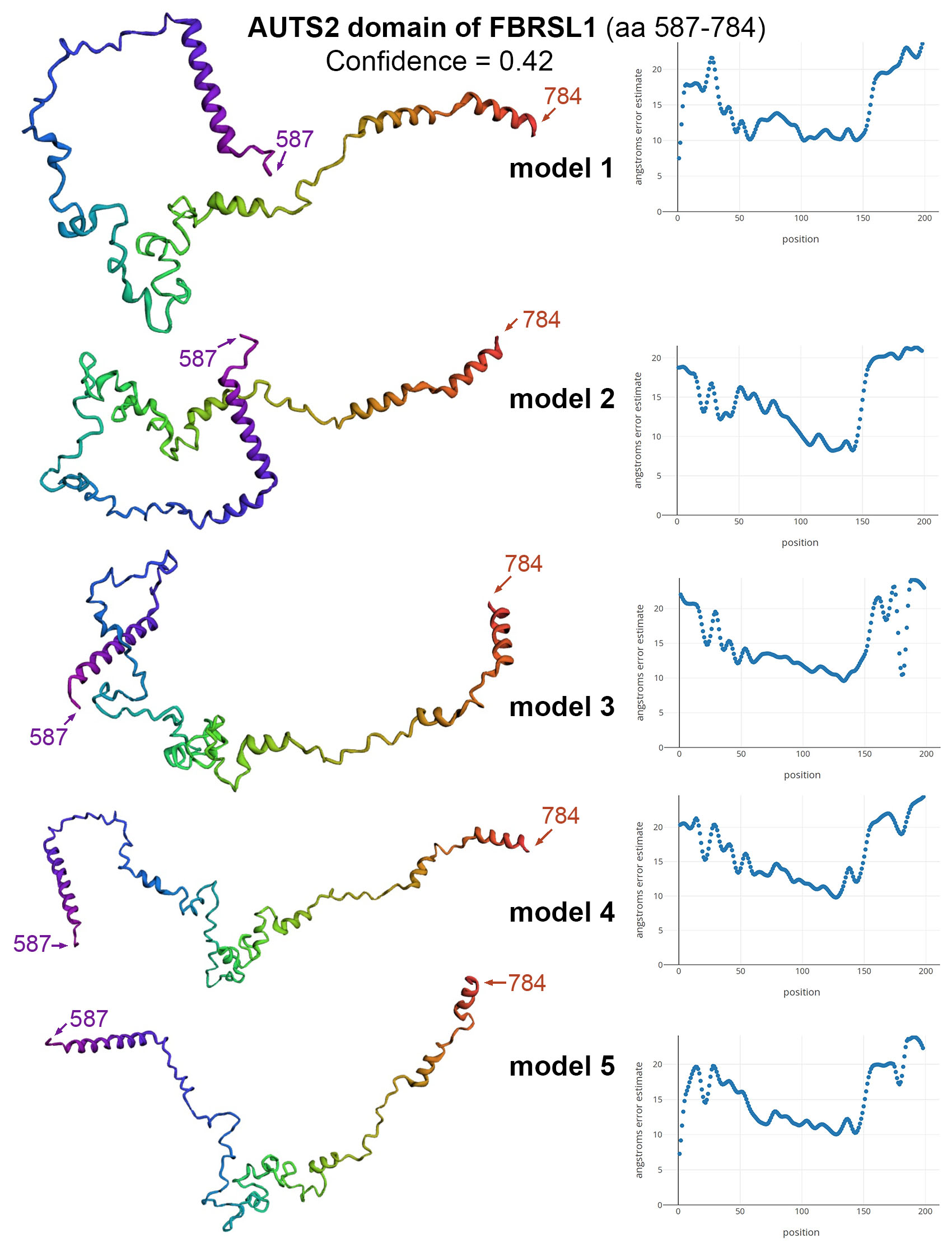

Supplement: Supplementary Figure 5 — Predictive modeling of the AUTS2 domain of FBRSL1 protein. The AUTS2 domain of FBRSL1 protein (aa 587–784) was modeled using RoseTTAfold. Left panels show predicted structures, and right panels show error estimates for each aa position, for models 1–5. Overall confidence was low (0.42 on scale 0–1), most likely because no structures of similar sequences were available. All of the models show stretches of disordered secondary structure with intervening alpha helices. Models not shown to the same scale. [file Image_5.JPEG]
